# NEW MODELS OF CARE FOR LONG-TERM CARE FACILITIES: WHAT DOES THE EVIDENCE TELL US?

**DOI:** 10.1093/geroni/igz038.909

**Published:** 2019-11-08

**Authors:** Katie Brittain, Dawn Craig, Karen Spilsbury, Paul Wilson, Katie Brittain, John Vines

**Affiliations:** 1 Northumbria University, Newcastle, United Kingdom; 2 University of Newcastle, Newcastle upon Tyne, England, United Kingdom; 3 University of Leeds, Leeds, England, United Kingdom; 4 University of Manchester, Manchester, England, United Kingdom; 5 Northumbria University, Newcastle upon Tyne, England, United Kingdom

## Abstract

Models of care are evolving to meet the demands of an ageing population in long-term care facilities. Syntheses of current evidence are an essential to inform future change. In this project we conducted a rapid synthesis of evidence relating to enhancing health in long term care facilities across technology and evaluation. Mapping reviews were conducted on the uses, benefits and challenges of technology in care homes and approaches to evaluation of new models of care. Systematic evidence syntheses addressed the questions of which technologies have a positive impact on resident health and well-being and which measurement tools have been validated for use in UK care homes. Key findings will be presented in animated format, including that the most promising interventions appear to be games that promote physical activity and enhance mental health. This presentation will highlight the benefits and importance of evidence synthesis to the development of models of care.

